# Epiploic Appendagitis Causing Small Bowel Obstruction: A Pleasant Surprise

**DOI:** 10.1155/2020/3126495

**Published:** 2020-07-04

**Authors:** Theodoros Hadjizacharias, Dionysios Dellaportas, Despoina Myoteri, Constantinos Nastos, Stavros Chaniotis, George Polymeneas

**Affiliations:** ^1^2nd Department of Surgery, Aretaieion University Hospital, Medical School, National and Kapodistrian University of Athens, Greece; ^2^Pathology Department, Aretaieion Hospital, Medical School, National and Kapodistrian University of Athens, Greece; ^3^3rd Department of Surgery, National and Kapodistrian University of Athens, Attikon University Hospital, Athens, Greece; ^4^Pediatric Department, General Hospital of Nikaia “Agios Panteleimon”, Greece

## Abstract

**Introduction:**

Epiploic appendagitis is a rare cause of acute abdominal pain, mimicking acute diverticulitis or appendicitis. Epiploic appendagitis causing small bowel obstruction is highly unusual, and only a handful of such cases have been reported so far. *Case Report*. A 69-year-old man presented with diffuse abdominal pain and vomiting over the last 12 hours. Clinical examination, laboratory, and imaging investigations showed small bowel obstruction, and after 12 hours of conservative management, surgical exploration was decided. During surgery, a dilated terminal ileum bowel loop was found densely adhered to the sigmoid colon, attached to an inflamed epiploic appendix. The small bowel was mobilized and freed, and the inflamed epiploic appendix was resected. The postoperative course was uneventful, and the patient was discharged on the 5^th^ postoperative day.

**Conclusion:**

Epiploic appendagitis, although a rare clinical entity, may explain small bowel obstruction symptoms.

## 1. Introduction

Small bowel obstruction (SBO) is a common cause of hospital admission, caused mostly in adults from previous surgery adhesions, neoplasms, or abdominal wall hernias [1]. On the other hand, epiploic appendagitis (EA) is a rare clinical condition believed to occur because of ischemic infarction due to torsion or thrombosis of the vein draining the epiploic appendix [2]. EA presents mostly as acute abdominal pain which can mimic acute diverticulitis or acute appendicitis, leading even to unnecessary laparotomies [3]. EA causing SBO has been reported only in a handful of cases to the best of our knowledge [4, 5]. A striking case of SBO due to EA is presented herein, emphasizing management dilemmas in such a clinical scenario.

## 2. Case Presentation

**Figure 1:**
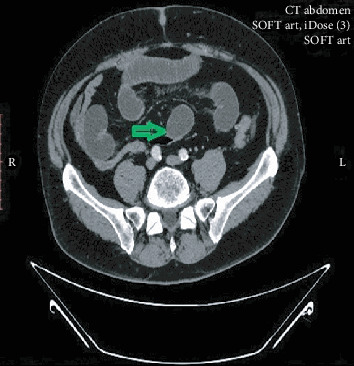
CT scan of the abdomen—green arrow showing the transition point in the terminal ileum, adhered to the sigmoid colon.

**Figure 2:**
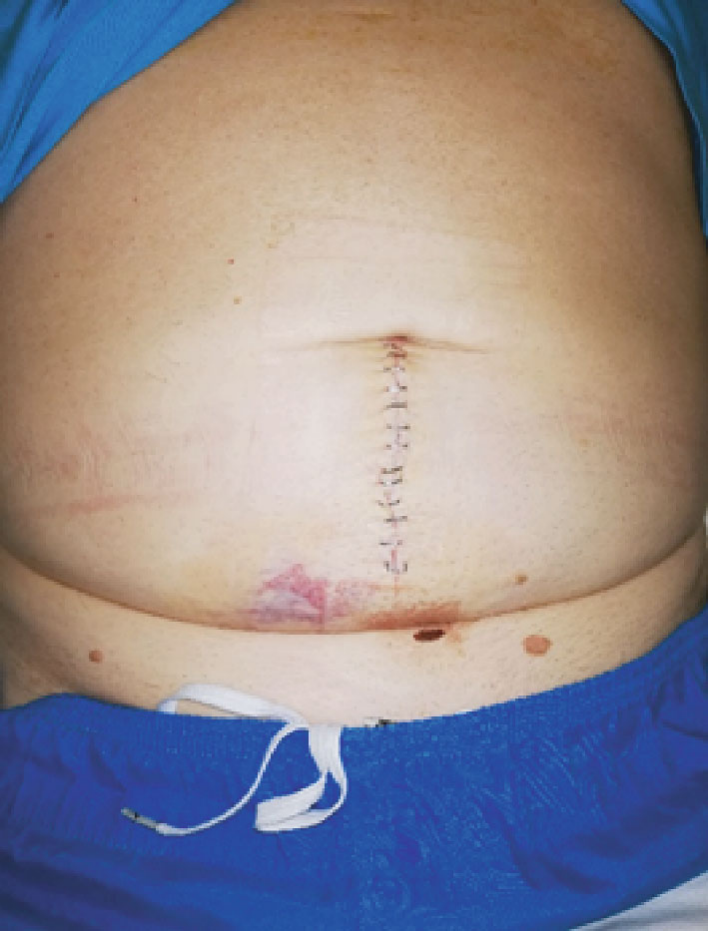
Lower midline laparotomy on the 4^th^ postoperative day.

**Figure 3:**
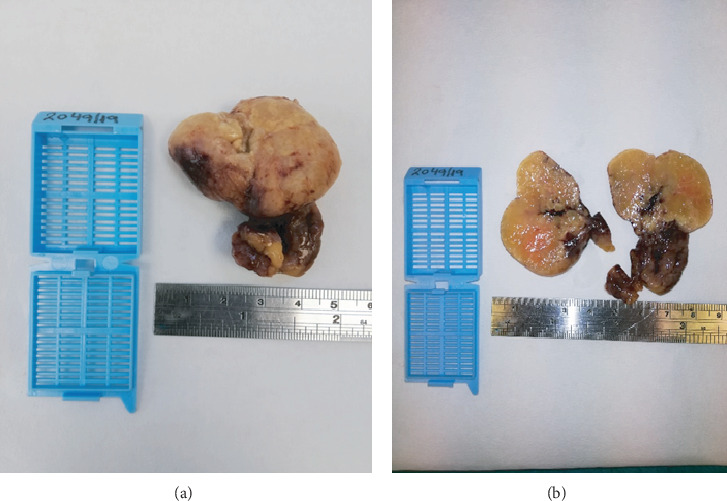
(a, b) Macroscopic view of the inflamed epiploic appendix after formalin fixation.

A 69-year-old man presented with colicky abdominal pain, flatulence, vomiting, and abdominal distension developed over the last 12 hours. The patient had arterial hypertension from his past medical history, while from his surgical history, he had underwent laparoscopic cholecystectomy 10 years earlier, and his BMI was 34. On clinical examination, he was mildly febrile with a temperature of 37.8°C, hemodynamically stable, and with marked abdominal distension and elevated inflammatory markers: white cell count 15000/78% neutrophilic type and CRP: 9. Otherwise, his laboratory tests were unremarkable. Abdominal X-ray showed dilated small bowel loops with air-fluid levels. Abdominal computed tomography (CT scan) followed and revealed SBO, showing a transition point in the terminal ileum (Figure 1). Abdominal pain was not resolving, and the patient remained febrile, despite conservative management with nasogastric tube placement and intravenous fluids. Exploratory laparotomy was decided starting with a lower midline incision (Figure 2). A dilated terminal ileum loop was revealed densely adhered to the sigmoid colon, creating an internal hernia-like condition, with the proximal small bowel protruding towards the pelvis being trapped and showing initial signs of reduced blood supply. At the transition point to the sigmoid colon, a 5 cm long, inflamed complexion was found initially thought to be an inflamed appendix. Surprisingly, it was found that the inflammatory mass was epiploic appendagitis. After mobilizing the adhered small bowel, the inflamed epiploic appendix was ligated at its base and resected, while normal colour and texture of the previously entrapped bowel loops were restored with the aid of warm normal saline. Histolopathological examination revealed hypertrophic epiploic appendagitis, with areas of necrosis and inflammation and hemorrhagic infiltration at peripheral sites (Figures 3(a) and 3(b)). The patient had an uneventful postoperative course and was discharged on the 5^th^ postoperative day.

## 3. Discussion

Epiploic appendages are pedunculated fat-containing structures arising from the large intestine wall, mostly developed on the transverse and sigmoid colons [6]. The size of an epiploic appendage ranges from 0.5 to 5 cm and is related to obesity [7]. Branches of a circular end-artery and a central draining vein compose their vasculature [8]. The exact pathophysiological role of the appendages remains unknown. Estimated incidence of EA is up to 8.8% per million people per year [9], and it is thought to arise due to vascular supply obstruction, caused by torsion or thrombosis.

Clinical presentation resembles acute diverticulitis or acute appendicitis, with left or right lower quadrant abdominal pain, which is more acute in onset though, with concurrent nausea, anorexia, and mild peritonism signs [10]. Laboratory findings are usually unhelpful, and an accurate diagnosis can be made only with the aid of abdominal CT scan [11]. Interestingly a centrally based high-attenuation focus within the inflamed epiploic appendage seen on CT has been reported as “the central dot sign” [12], which can help with the correct diagnosis. Treatment options include anti-inflammatory medication and outpatient management in most cases with supportive care, while misdiagnosis is very common leading to unnecessary hospital admissions, antibiotic use, or even unnecessary surgery [13].

As mentioned above, 90% of SBO cases are caused by adhesions, hernias, and neoplasms [1], while unusual causes as inflammatory bowel disease complications, intussusception, postischemic stenosis, endometriosis, postradiation therapy strictures, anastomotic bowel strictures, gallstones, foreign bodies, phytobezoars, and tuberculosis account for the remainder 10% [14]. An abdominal inflammatory condition can cause ileus; however, in the case presented above, EA caused mechanical SBO, and this particular clinical scenario is very seldom. From the surgeon's point of view, it is favorable and technically less demanding to resolve than usual SBO operations.

In conclusion, EA should be included in the differential diagnosis list of unusual SBO causes.

## Data Availability

None.
